# Age at diagnosis of inflammatory bowel disease influences early development of colorectal cancer in inflammatory bowel disease patients: a nationwide, long-term survey

**DOI:** 10.1007/s00535-012-0603-2

**Published:** 2012-05-25

**Authors:** J. E. Baars, E. J. Kuipers, M. van Haastert, J. J. Nicolaï, A. C. Poen, C. J. van der Woude

**Affiliations:** 1 Department of Gastroenterology and Hepatology, Erasmus MC, ‘s Gravendijkwal 230, Room Ba 393, 3015 CE Rotterdam, The Netherlands; 2 Department of Internal Medicine, Erasmus MC, Rotterdam, The Netherlands; 3 Department of Gastroenterology and Hepatology, Martini Hospital, Groningen, The Netherlands; 4 Department of Gastroenterology and Hepatology, Haga Hospital, The Hague, The Netherlands; 5 Department of Gastroenterology and Hepatology, Isala Clinics, Zwolle, The Netherlands

**Keywords:** Ulcerative colitis, Crohn’s disease, Colorectal carcinoma, Risk factor

## Abstract

**Background:**

Data on clinical characteristics of patients with inflammatory bowel disease (IBD)-related colorectal cancer (CRC) are scarce and mainly originate from tertiary referral centres. We studied patient and disease characteristics of IBD-related CRC in a nationwide IBD cohort in general hospitals. Main outcome parameters were time to develop CRC, and factors associated with early CRC development.

**Methods:**

All IBD patients diagnosed with CRC between 1 January 1990 and 1 July 2006 were identified using a nationwide automated pathology database (PALGA). Patient charts were assessed to confirm diagnosis and collect clinical data. Early CRC was defined as CRC diagnosed less than 8 years after IBD diagnosis. Statistical analysis was performed using descriptive statistics, independent *t* tests, binary logistic regression and Cox-regression analysis.

**Results:**

Diagnosis of IBD-related CRC was confirmed in 251 patients (171 ulcerative colitis, 77 Crohn’s disease, 3 unclassified colitis), 161 males (64 %). Median time from IBD diagnosis to CRC diagnosis was 12 years (IQR 4–20); 89 patients (35 %) developed early CRC. Type of IBD, gender, concomitant PSC, pseudopolyps, extent of inflammation, and medication use were not related to early CRC (*p* > 0.05). IBD diagnosis at older age (HR for 10 years older age 2.25; 95 % CI 1.92–2.63) was related to early CRC. Twenty-three patients (12 %) had been included in a surveillance programme prior to CRC diagnosis. Patients in the surveillance group had a significantly better tumor stage (*p* = 0.004).

**Conclusions:**

We emphasize the problem of a high proportion of IBD-associated CRCs developing before the recommended start of surveillance. Therefore, we suggest that older age at IBD onset could be an additional factor to start surveillance in IBD patients.

## Introduction

Patients with chronic colitis due to ulcerative colitis (UC) or Crohn’s disease (CD) carry an increased risk for colorectal carcinoma (CRC) [1–3]. Previous studies have mainly focused on the risk of acquiring inflammatory bowel disease (IBD)-related CRC [4–9]. However, data on clinical characteristics of IBD-related CRC cohorts, including factors that lead to earlier development of CRC, are scarce. Available studies have particularly focused on the relationship between age at onset of IBD and the interval to CRC development [10–12]. However, the results of these studies were inconsistent. Moreover, most studies focussing on the interval between IBD and CRC are based on specific tertiary cohorts including patients with a more severe and complicated disease [11, 13, 14]. Data on both patient and disease characteristics of patients with IBD-related CRC from non-referral, community care centres are lacking. It is therefore unknown whether the clinical characteristics of these high-risk tertiary referrals differ from the average IBD population in general hospitals.

Recently, the characteristics of patients with IBD-related CRC from academic medical centres in the Netherlands have been published [14]. Approximately 20 % of all IBD-related CRCs in this cohort occurred in the first decade after onset of IBD. The cohort of 149 patients with IBD-related CRC mainly consisted of male UC patients with extensive disease. This was a selected population with results which may not reflect the total IBD population. Furthermore, the study did not report any risk factors that influence the interval between IBD and CRC in this academic population, and thus did not allow identification of any subgroup of IBD patients which would require earlier start of CRC surveillance.

Surveillance colonoscopies are an important strategy to detect CRC at an early stage and thereby decrease CRC-related morbidity and mortality [15, 16]. Currently, CRC surveillance is recommended to start 8–10 years after the onset of IBD in patients with extensive colitis and 15–20 years in those with left-sided colitis [17, 18]. More recent guidelines recommend a first screening colonoscopy 8–10 years after disease onset in all IBD patients irrespective of disease extent [19, 20].

To implement evidence based surveillance strategies in the total IBD population, characteristics of patients with IBD-related CRC in general hospitals are needed. Moreover, parameters which lead to early development of CRC in general hospitals need to be determined to identify patients in need of earlier start of surveillance. For that reason, our aim was to assess patient characteristics and distinctive disease characteristics of IBD-related CRC in patients from general hospitals in the Netherlands.

## Materials and methods

### Study population

Inflammatory bowel disease-related CRC patients in all 93 general hospitals in the Netherlands were identified using the nationwide network and registry of histo- and cytopathology (PALGA) [21]. The PALGA database contains all pathology reports generated in the Netherlands from 1990 until present. These reports are all concluded with diagnostic terms in line with SNOMED^®^ terminology. Each subject in the database has a unique identifier which allows tracking of individual patients over time and throughout the country. The following search criteria were used: (colon AND/OR all primary carcinoma, colon AND/OR all carcinoma in situ, colon AND/OR all micro invasive tumors, rectum AND/OR all primary carcinoma, rectum AND/OR all carcinoma in situ, rectum AND/OR all micro invasive tumors) AND (colitis AND/OR ulcerative colitis AND/OR indeterminate colitis AND/OR idiopathic colitis AND/OR Crohn’s disease).

Patients with histologically confirmed IBD and a synchronous or metachronous CRC diagnosed between January 1, 1990 and July 1, 2006 were included. All IBD patients diagnosed with colorectal cancer in a general hospital were included, irrespective of where they were treated afterwards. Patients who were initially treated and diagnosed with cancer in an academic hospital and referred to a general hospital after cancer diagnosis were excluded. Clinical data were needed to confirm the diagnosis of IBD-related CRC.

The study was approved by the Institutional Review Board of the Erasmus MC Rotterdam, the Netherlands.

### Data extraction

Further clinical data could be collected from 78 hospitals in the Netherlands. The results from the search were verified by the treating physicians in each hospital as well as by an external expert. Clinical data were collected from the patient charts, endoscopy reports and pathology reports. The following data were collected: type of IBD, age, gender, date of diagnosis of IBD and CRC, date of onset symptoms attributable to IBD, duration of disease, disease characteristics of IBD and CRC, history of colonic surgery, presence of concomitant PSC, use of medication, and surveillance details. Colonoscopies in the setting of dysplasia screening with multiple biopsies according to the surveillance guidelines were counted as surveillance colonoscopy [17, 18]. The complete medical history was assessed, including prior colonoscopies and pathology reports. Extent of disease was subdivided in five categories based on type and extent of inflammation: left-sided UC, extensive UC, limited CD, extensive CD or unclassified colitis. Limited CD was defined as <50 % segmental CD including those with terminal ileitis or ileocoecal inflammation. Extensive CD was defined as >50 % segmental CD. Severity of disease was graded as mild, moderate or severe colitis based on both histological and endoscopic features. Duration of medication-use during follow-up was divided into four categories (0–25, 25–50, 50–75, >75 % of duration of follow-up). Patients who appeared to have chronic inflammation due to any other cause but IBD, as well as those in whom a diagnosis of CRC had not been confirmed histologically, were excluded for further analysis. “Early CRC” was defined as CRC diagnosed within 8 years after IBD diagnosis, irrespectively of disease extent.

### Statistical analysis

Statistical analysis was performed using descriptive statistics, independent samples *t* tests, logistic regression, and Cox-regression analysis. Follow-up time was defined as time in years from start of diagnosis of IBD to diagnosis of CRC. Patients who were simultaneously diagnosed with IBD and CRC were excluded for all risk factor analyses for the interval between IBD and CRC, since no comments can be made on the interval between IBD and CRC in these patients. Cox’s proportional hazards regression was used to estimate univariable and multivariable hazard ratios to analyse the effect of several risk factors for early development of IBD-related CRC. Multivariate Cox regression was performed stepwise-backward in which all variables with a *p* value below 0.10 were included, provided they had a plausible sign [22]. Univariate and multivariate logistic regression was used to identify clinical characteristics related to early CRC (<8 years after IBD diagnosis). Groups were compared based on type of IBD, gender, presence of concomitant PSC, pseudopolyps, rectal sparing, severity and extent of inflammation, medication use (ever used and duration of use), family history of CRC, and histological signs of dysplasia prior to diagnosis of CRC. In a sub-analysis we excluded patients ≥65 years old to minimize the interference of sporadic CRC. Multivariate logistic regression was performed stepwise-backward in which all variables with a *p* value below 0.10 were included. Statistical analysis was performed with SPSS for Windows software (version 15.0).

## Results

### PALGA search

**Fig. 1:**
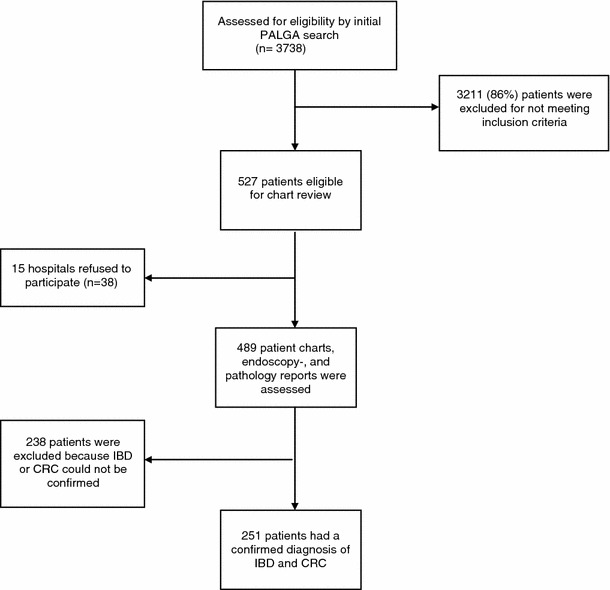
Flow chart study population

Figure 1 represents a flow chart of the study population. The initial search in the PALGA system revealed 3738 patients with a diagnosis compatible with IBD-associated CRC (1941 males, 1797 females). After further analysis of the pathology excerpts within the PALGA search, 3211 (86 %) patients were excluded for reasons of lack of histological confirmation of IBD or CRC, colonic inflammation diagnosed years after the diagnosis of CRC, or colitis due to other causes than IBD (e.g. diverticulitis).

Of the remaining 527 patients with a possible diagnosis of IBD-related CRC, 59 patients (11 %) were >65 years old. This group consisted of 37 males and 22 females, of whom 51 patients had UC and 8 patients had CD.

In total, 527 patients were therefore eligible for detailed chart review to confirm the diagnosis of IBD-related CRC. Fifteen hospitals with a total of 38 (7 %) patients were not willing to participate in the detailed chart review.

In total, 489 patient charts, endoscopy and pathology reports were assessed to confirm the diagnosis of IBD-related CRC and collect clinical data. In 251 (51 %) out of these 489 patients, the histological suspicion of IBD-related CRC was confirmed based on additional clinical data, including endoscopy and full histology reports.

### Patient characteristics

**Fig. 2:**
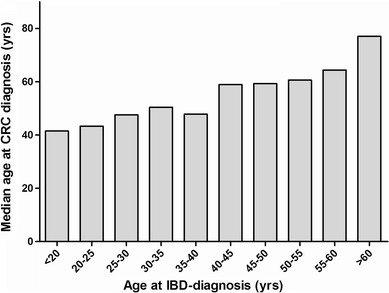
Age at IBD diagnosis versus age at CRC diagnosis. The age at IBD diagnosis is plotted against the age at which patients are diagnosed with CRC. This figure demonstrates that age at onset of IBD is not related to the age at which patients develop CRC

Overall, in 251 patients the diagnosis of IBD-related CRC was confirmed. Of these patients, 161 were male (64 %), 171 had UC (68 %), 77 had CD (31 %) and three patients had unclassified colitis (2 %). Median age [interquartile range (IQR)] at diagnosis of IBD was 40.1 years (IQR 24–58). Median age at CRC diagnosis was 56.4 years (IQR 44–65). Figure 2 shows the age at CRC diagnosis plotted against the age at IBD diagnosis. Additional case characteristics are listed in Table 1. In 170 (67 %) out of 251 cases start of IBD complaints was not retrievable from the charts. Twenty-three patients (12 %) had been included in a dysplasia surveillance program prior to CRC diagnosis [data on surveillance were missing in 63 patients (25 %)]. On average, patients were included in a dysplasia surveillance programme after 10 years of disease (IQR 7.5–24) with an interval between each surveillance colonoscopy of 1 year (IQR 1–2.3).

**Table 1:** Patient characteristics

	*N* All cases (%)			*N* Simultaneously diagnosed IBD + CRC excluded (%)			
	Total	UC	CD	Total		UC	CD
No. of patients	251	171	77	216		150	64
Disease							
Ulcerative colitis	171 (68)	171	–	150 (69)		150	–
Crohn’s disease	77 (31)	–	77	64 (30)		–	64
Unclassified colitis	3 (2)	–	–	2 (1)		–	–
Gender							
Male	161 (64)	114 (67)	45 (58)	138 (64)		103 (69)	34 (53)
Female	90 (36)	57 (33)	32 (42)	78 (36)		47 (31)	30 (47)
Median interval between IBD and CRC (IQR)	11.7 years (IQR 4–21)	11 years (IQR 4–20)	13.6 years (IQR 4–25)	14.0 years (IQR 7–23)		13.2 years (IQR 6–22)	17.2 years (IQR 9–26)
Median age at diagnosis IBD (IQR)	41.1 years (IQR 24–58)	45.7 years (IQR 26–64)	32.6 years (IQR 23–51)	35 years (IQR 23–56)		43 years (IQR 24–60)	28.8 years (IQR 21–42)
Median age at diagnosis CRC (IQR)	56.4 years (IQR 44–65)	58.4 years (IQR 46–69)	50.7 years (IQR 41–61)	56.4 years (IQR (43–65)		58.9 years (IQR 47–70)	50 years (IQR 40–60)
Disease extent							
Leftsided colitis (UC)	23 (9)	22 (13)	–	17 (8)		17 (11)	–
Pancolitis (UC)	57 (23)	79 (46)	–	54 (25)		54 (36)	–
<50 % segmental colitis (limited CD)	23 (9)	–	20 (26)	15 (7)		–	15 (23)
>50 % segmental colitis (extensive CD)	38 (15)	–	34 (44)	35 (16)		–	34 (53)
Extensive unclassified colitis	2 (1)	–	–	1 (0)		–	–
Unknown	108 (40)	70 (410)	23 (30)	94 (44)		79 (53)	15 (23)
Severity of disease							
Mild	20 (8)	15 (9)	4 (5)	15 (7)		11 (7)	4 (6)
Moderate	62 (25)	38 (22)	23 (30)	56 (26)		35 (23)	20 (31)
Severe	73 (29)	48 (29)	25 (32)	65 (30)		43 (29)	22 (34)
Unknown	96 (38)	70 (41)	25 (32)	80 (37)		61 (41)	18 (28)
Pseudopolyps							
Yes	72 (29)	51 (30)	29 (38)	71 (33)		45 (30)	25 (39)
No	81 (32)	45 (26)	25 (32)	64 (30)		41 (27)	22 (34)
Unknown	98 (40)	75 (44)	23 (30)	81 (38)		64 (43)	17 (27)
Concomitant PSC							
Yes	19 (8)	14 (8)	4 (52)	18 (8)		13 (9)	4 (6)
No	162 (65)	101 (59)	59 (77)	140 (68)		90 (60)	49 (77)
Unknown	70 (28)	56 (33)	14 (18)	58 (27)		47 (31)	11 (17)
Positive family history CRC							
Negative	82 (33)	53 (31)	29 (38)	68 (33)		44 (29)	23 (36)
Positive 1st degree	9 (4)	5 (3)	5 (6)	7 (3)		5 (3)	2 (3)
Positive 2nd degree	4 (2)	1 (1)	1 (1)	4 (2)		3 (2)	1 (2)
Unknown	156 (62)	112 (63)	42 (54)	137 (63)		98 (65)	38 (59)
Ongoing active inflammation							
No	125 (50)	85 (50)	39 (51)	115 (53)		79 (53)	35 (55)
Yes	38 (15)	23 (13)	14 (18)	28 (13)		17 (11)	11 (17)
Unknown	88 (35)	63 (37)	24 (31)	73 (34)		54 (36)	18 (28)
Colon surgery prior to onset IBD							
No	165 (66)	104 (61)	58 (75)	143 (66)		93 (62)	48 (75)
Yes	7 (3)	3 (2)	4 (5)	7 (3)		3 (2)	4 (6)
Unknown	79 (31)	64 (37)	15 (19)	66 (31)		54 (36)	12 (19)
Rectal sparing (UC + unclassified)							
Negative	73 (43)	70 (41)	–	62 (41)		60 (40)	–
Positive	3 (2)	3 (2)	–	3 (2)		3 (2)	–
Unknown	95 (56)	98 (570)	–	87 (57)		87 (58)	–
Dysplasia found prior to CRC diagnosis							
No	164 (65)	99 (58)	63 (82)	139 (64)		87 (58)	51 (80)
Yes	30 (12)	23 (13)	6 (8)	28 (13)		21 (14)	6 (9)
Unknown	57 (23)	49 (29)	8 (10)	49 (23)		42 (28)	7 (10)

### Tumor characteristics

Forty-seven percent of tumors were located in the rectum (*n* = 54) or sigmoid (*n* = 40). In 73 % of the cases (*n* = 143) the tumor occurred in the colon segment affected by colitis. The location of chronic inflammation did not influence the location of the tumor, *p* = 0.17. For UC patients, those with left-sided UC had a RR of 1.5 (95 % CI 0.51–4.3, *p* = 0.5) to develop a tumor in the right part of the colon compared with patients with pancolitis. Moreover, eight (35 %) out of 23 patients with a known left-sided UC developed a tumor in the right part of the colon.

Eighty-four patients (43 %) had T3 tumors and 31 (16 %) metastases at time of CRC-diagnosis. Additional tumor characteristics are listed in Table 2. The distribution in tumor sites differed between UC and CD patients. For 59 (24 %) patients (50 UC and 9 CD), the exact tumor location could not be retrieved. In the remainder, 78 out of 121 UC patients (64 %) developed a tumor in the left part of the colon, compared with 33 out of 68 CD patients (49 %), *p* = 0.022. Patients who were included in a surveillance programme had a significantly better AJCC tumor stage (*p* = 0.004). Twelve (52 %) out of 23 patients (AJCC tumor stage unknown in one patient) in the surveillance group had a stage I tumor compared with 31 (19 %) out of 165 patients in the non-surveillance group (AJCC tumor stage unknown in eight patients). In addition, surveillance was associated with a better T-stage (*p* = 0.002). Only five patients in the surveillance group (22 %) had a T3 tumor at time of diagnosis compared to 67 patients (41 %) in the non-surveillance group. Surveillance did not influence the metastasis at time of diagnosis of during follow-up (*p* = 0.9).

**Table 2:** Tumor characteristics

	*N* All cases (%)			*N* Excluding simultaneously diagnosed IBD + CRC (%)		
	Total	UC	CD	Total	UC	CD
No. of patients	251	171	77	216	150	64
CRC location						
Rectum	54 (22)	35 (20)	18 (23)	45 (21)	28 (19)	17 (27)
Sigmoid	40 (16)	29 (17)	10 (13)	36 (17)	26 (17)	9 (14)
Descending colon	9 (4)	8 (5)	1 (1)	9 (4)	8 (5)	1 (2)
Splenic flexure	10 (4)	6 (4)	4 (5)	8 (4)	4 (3)	4 (6)
Transverse colon	17 (7)	8 (5)	9 (12)	15 (7)	8 (5)	7 (11)
Hepatic flexure	6 (2)	3 (2)	3 (4)	6 (3)	3 (2)	3 (5)
Ascending colon	17 (7)	10 (6)	7 (9)	12 (6)	8 (5)	4 (6)
Caecum	27 (11)	14 (8)	12 (16)	22 (10)	13 (9)	8 (13)
Double tumors	10 (4)	7 (4)	3 (4)	10 (5)	7 (5)	3 (5)
Unknown	61 (24)	51 (30)	10 (13)	53 (5)	45 (30)	8 (13)
T-stage						
Tis	10 (4)	8 (5)	3 (4)	8 (4)	5 (3)	3 (5)
T1	25 (10)	19 (11)	5 (6)	20 (9)	16 (11)	4 (6)
T2	36 (14)	25 (15)	11 (14)	31 (14)	23 (15)	8 (13)
T3	84 (33)	54 (32)	30 (39)	72 (33)	46 (31)	25 (39)
T4	32 (13)	14 (8)	17 (22)	29 (13)	13 (9)	15 (23)
Tx	64 (25)	51 (30)	11 (14)	56 (26)	47 (31)	9 (14)
N stage						
N0	102 (41)	63 (37)	38 (50)	85 (39)	53 (35)	32 (50)
N1	62 (25)	39 (23)	22 (29)	55 (25)	36 (24)	18 (28)
N2	20 (8)	13 (8)	6 (8)	18 (8)	12 (8)	5 (8)
Nx	65 (26)	56 (33)	11 (14)	58 (27)	49 (33)	6 (15)
M stage						
M0	155 (62)	100 (58)	53 (69)	129 (60)	85 (57)	43 (67)
M1	32 (13)	20 (11)	12 (16)	32 (15)	19 (13)	12 (19)
Mx	64 (26)	51 (30)	12 (16)	55 (25)	46 (31)	9 (15)
AJCC stage (6th edition)						
I	45 (18)	(18)	13 (17)	37 (17)	27 (18)	10 (16)
IIa	32 (13)	20 (12)	12 (16)	27 (13)	17 (11)	10 (16)
IIb	6 (2)	3 (2)	3 (4)	4 (2)	2 (1)	2 (3)
IIIa	13 (5)	12 (7)	1 (1)	11 (5)	11 (7)	–
IIIb	35 (14)	18 (11)	16 (21)	31 (14)	16 (11)	14 (22)
IIIc	10 (4)	8 (5)	2 (3)	8 (4)	7 (47)	1 (2)
IV	34 (14)	19 (11)	14 (18)	33 (15)	19 (13)	13 (21)
Unknown	76 (30)	60 (35)	16 (21)	65 (30)	51 (34)	14 (22)
Metastasis spread during follow-up	59 (24)	32 (19)	25 (32)	54 (25)	31 (21)	21 (14)
CRC in previous inflamed areas	143 (57)	89 (52)	52 (68)	129 (60)	81 (54)	47 (73)

### CRC development within 8 years after IBD diagnosis

**Fig. 3:**
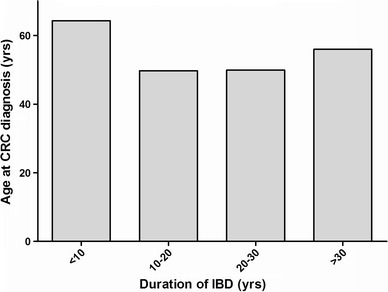
Age at CRC diagnosis is independent of duration of IBD. The duration of IBD is plotted against the median age at which patients developed CRC. This figure demonstrates that age at CRC-diagnosis is independent of duration of IBD

In seven (2.8 %) patients the exact date at which they had been diagnosed with IBD was unknown. These patients were therefore excluded for the following analyses with regard to the interval between onset of IBD and CRC diagnosis. The time interval between IBD and CRC with entry point confirmed diagnosis of IBD was, on average, 12 years (IQR 4–20). Overall, 89 patients (35 %) developed CRC within 8 years after IBD diagnosis, and 115 patients (47 %) experienced CRC within the first decade after IBD diagnosis. In the group that developed CRC within 8 years after diagnosis of IBD, the median age at IBD diagnosis was 58.8 years (IQR 48–69), compared with a median age of 28.4 years (IQR 22–45) in those who developed CRC > 8 years of IBD, *p* < 0.001. The age at CRC diagnosis was on average 61.6 years (IQR 49–73) in those who developed CRC < 8 years of disease, compared with a median age of 53.5 (IQR 43–62) in those who developed CRC > 8 years of disease (*p* = 0.08). Figure 3 shows that the median age at CRC diagnosis is independent of the duration of IBD. Table 3 presents the clinical characteristics of those who developed CRC within 8 years. Excluding patients >65 years old did not influence the results with regard to early development of CRC.

**Table 3:** One third of patients developed CRC within 8 years after IBD diagnosis: clinical characteristics of early CRC

	Early CRC < 8 years (%)	*p* value (univariate logistic regression)	RR (95 % CI) (multivariate logistic regression)	*p* value (multivariate logistic regression)	
Total no. of patients with early CRC	89 (35)				
Type of IBD		*p* = *0.3*		–	
Ulcerative colitis	64 (37)				
Crohn’s disease	23 (30)				
Unclassified colitis	2 (67)				
Gender		*p* = *0.8*		–	
Male	58 (36)				
Female	31 (34)				
Age at diagnosis IBD (years)		*p* **<** *0.001*	*HR 10* *years older age: 4.5 (2.7* **–** *7.2)*	*p* *<* *0.001*	
<20	1 (3)				
20–30	3 (6)				
30–40	9 (28)				
40–50	14 (40)				
50–60	19 (56)				
>60	42 (75)				
Disease extent (years)		*p* *=* *0.005*		*p* = *0.07*	
Left-sided colitis (UC)	11 (48)				
Pancolitis (UC)	11 (20)				
<50 % segmental colitis (limited CD)	12 (52)				
>50 % segmental colitis (extensive CD)	5 (14)				
Extensive unclassified colitis	2 (100)				
Severity of disease		*p* **=** *0.01*		*p* = *0.25*	
Mild	13 (62)				
Moderate	17 (27)				
Severe	18 (25)				
Pseudopolyps		*p* = *0.06*		–	
No	30 (38)				
Yes	17 (24)				
Concomitant PSC		*p* = *0.7*		–	
No	49 (31)				
Yes	5 (26)				
Positive family history CRC		*p* = *0.98*		–	
Negative	30 (37)				
Positive 1st degree	3 (3)				
2nd degree	–				
Ongoing active inflammation		*p* **=** *0.01*	*5.2 (1.6*–*16.3)*	*p* *=* *0.018*	
No	30 (24)				
Yes	18 (47)				
Colon surgery prior to onset IBD		*p* = *0.11*		–	
No	45 (28)				
Yes	4 (57)				
Rectal sparing (UC)		*p* = *0.25*		–	
Negative	23 (32)				
Positive	2 (67)				

### Simultaneous diagnosis of IBD and CRC

Thirty-five patients (14 %) were diagnosed with IBD and CRC simultaneously of within 6 months after IBD diagnosis (21 had UC, 13 had CD, and one patient had unclassified colitis). Of these 13 CD patients one had a tumor in the rectum, one in the sigmoid, two in colon transversum, four in the ascending colon, four in the caecum (data missing in one patient). For the UC patients, in eight patients the tumor was located in the rectum, in five patients in the sigmoid, two in the splenic flexure, two in the ascending colon and one in the caecum. Patient characteristics of this group are listed in Table 1 and tumor characteristics in Table 2. All these patients were >37 years old at time of IBD diagnosis. One patient reported having had IBD-related complaints for 23 years from the age of 19 onwards (data on start of symptoms missing in 21 patients). Of the CD patients with a simultaneous diagnosis of IBD and CRC, seven had limited CD (78 %) against two patients with extensive CD (22 %) (*p* = 0.022). In four patients data on the extent of CD was missing. Of the 36 UC patient diagnosed simultaneously with IBD and CRC, in six patients the extent of IBD was known: three had left-sided colitis and three had pancolitis (*p* = 0.35).

### Factors influencing the time interval between onset of IBD and CRC

As stated in the methods section, patients with simultaneous diagnosis of IBD and CRC were excluded for the following risk factor analysis with regard to the time interval between IBD and CRC.

Univariable cox regression analysis showed that type of IBD, gender, presence of concomitant PSC, pseudopolyps, rectal sparing (UC patients), severity and extension of inflammation, a positive family history of CRC, presence of dysplasia prior to CRC diagnosis, or ongoing active inflammation of IBD, without any remission during follow-up, were not significantly associated with early development of CRC (all *p* > 0.05, Table 4). Diagnosis of IBD at older age was associated with early development of CRC (HR for 10 years older age 2.25; 95 % CI 1.92–2.63). Excluding patients >65 years old did not influence the factors associated with early development of CRC.

The extent of inflammation did not correspond with the time to development of CRC (*p* = 0.53). However, patients with pancolitis had more often been treated with 5-ASA (49/50 = 98 %) than patients with left-sided colitis (15/20 = 75 %), *p* = 0.06. In addition, UC patients with left-sided colitis were more often diagnosed with IBD at an older age (median age 43.4 years; IQR 27–55) compared with UC patients suffering from pancolitis (median age 27.9 years; IQR 21–44), *p* = 0.02.

For all medications (5-ASA, corticosteroids, azathioprine, methotrexate, anti-TNF, ursodeoxycholic acid, folic acid and ferrofumerate) no significant correlations with the time to develop CRC were found (Table 5). Duration of medication use did not significantly influence the time to CRC diagnosis for any type of drug. Starting 5-ASA directly at the time of IBD diagnosis did not influence the interval between IBD and CRC (*p* = 0.24).

**Table 4:** Parameters influencing a short interval between IBD and CRC development (univariate cox regression)

	Median time to CRC-diagnosis in years (IQR)	Hazard ratio (95 % CI)	*p* value
No. of patients	215 (%)^a^		
Type of IBD			*p* = *0.3*
Ulcerative colitis	13.0 (6–21)	1.0	
Crohn’s disease	17 (9.3–26.8)	0.59 (0.15–2.38)	
Unclassified colitis	11 (5–17)	0.48 (0.12–2.0)	
Gender			*p* = *0.48*
Female	14 (6.5–22.0)	1.0	
Male	14 (7–24)	1.1 (0.83–1.47)	
Age at diagnosis IBD (years)			*p* < *0.001*
<20	24.5 (18.8–34.9)	1.0	
20–30	19.8 (15.4–30.7)	1.7 (1.1–2.7)	
30–40	16 (8.3–21.6)	3.9 (2.3–6.7)	
40–50	12.1 (4.5–14.9)	7.0 (4.0–12.2)	
50–60	7.2 (4.2–8.4)	21.0 (10.3–42.7)	
60–65	3.5 (0.9–4.8)	59.8 (22.1–161.7)	
Disease extent (years)			*p* = *0.3*
Left-sided colitis (UC)	11.5 (2.5–22.8)	1.0	
Pancolitis (UC)	17.7 (10.9–24.2)	0.66 (0.39–1.12)	
<50 % segmental colitis (limited CD)	12.7 (4.5–29.7)	0.73 (0.38–1.41)	
>50 % segmental colitis (extensive CD)	18.0 (13.4–25.8)	0.65 (0.37–1.13)	
Extensive unclassified colitis	4.3 (4.3–4.3)	5.41 (0.69–42.5)	
Severity of disease			*p* = *0.26*
Mild	9.0 (2.8–22.7)	1.0	
Moderate	18.9 (9.5–24.9)	0.62 (0.36–1.01)	
Severe	16.3 (8.5–22.2)	0.72 (0.42–1.24)	
Pseudopolyps			*p* = *0.7*
No	17.7 (6.1–26)	1.0	
Yes	16.0 (8–23.2)	1.07 (0.76–1.5)	
Concomitant PSC			*p* = *0.5*
No	16.6 (8.1–25.1)	1.0	
Yes	18.3 (8.2–23.9)	1.19 (0.72–1.95)	
Positive family history CRC			*p* = *0.19*
Negative	16.1 (7.5–23.2)	1.0	
Positive 1st degree	15.0 (10–20.3)	1.23 (0.56–2.7)	
2nd degree	32.5 (20.2–37.3)	0.43 (0.16–1.2)	
Ongoing active inflammation			*p* = *0.11*
No	17.4 (9.3–24.7)	1.0	
Yes	14.5 (3.8–23.2)	1.39 (0.93–2.07)	
Colon surgery prior to onset IBD			*p* = *0.08*
No	17.5 (9.7–24.7)	1.0	
Yes	7.3 (2.1–23.2)	1.97 (0.92–4.23)	
Rectal sparing (UC)			*p* = *0.08*
Negative	13.7 (7.5–20.8)	1.0	
Positive	7.7 (4.8–12.4)	2.91 (0.88–9.65)	
Dysplasia found prior to CRC diagnosis			*p* = *0.23*
No	15.4 (8.4–24.1)	1.0	
Yes	17.6 (5.4–24.6)	0.83 (0.61–1.13)	

^a^36 patients with simultaneous IBD and CRC were excluded for this risk factor analysis

**Table 5:** Medication use does not protect against early CRC

	Cases	Median time to diagnosis CRC in years (IQR)	Hazard Ratio (95 % CI)	*p* value
No. of patients	159 (%)^a^		–	
5-ASA				*p* = *0.69*
Yes	137 (86)	17.3 (9.8–24.2)	0.91 (0.58–1.44)	
No	22 (14)	16.4 (3.3–28.1)	1.0	
Thiopurines				*p* = *0.97*
Yes	36 (23)	17.4 (13.1–24.6)	1.01 (0.69–1.45)	
No	123 (77)	16.9 (8–24.5)	1.0	
Corticosteroids				*p* = *0.24*
Yes	102 (64)	17.6 (10.9–24.5)	0.82 (0.59–1.14)	
No	57 (36)	15.9 (5–24.6)	1.0	
MTX				*p* = *0.47*
Yes	2 (1)	14.7 (11.6–17.8)	1.68 (0.41–6.83)	
No	157 (99)	17.3 (8.8–24.5)	1.0	
Anti-TNF				*p* = *0.34*
Yes	4 (3)	17.2 (8.4–17.8)	1.63 (0.6–4.46)	
No	155 (97)	17.2 (9–24.5)	1.0	
Ascal				*p* = *0.34*
Yes	5 (3)	17.2 (5.3–34.5)	0.65 (0.26–1.6)	
No	154 (97)	15.4 (8.9–24.5)	1.0	
NSAIDS				*p* = *0.11*
Yes	7 (4)	14.7 (0.9–20.6)	1.85 (0.86–3.98)	
No	152 (96)	15.7 (9.1–24.6)	1.0	
Folic acid				*p* = *0.71*
Yes	16 (10)	14.6 (10.6–27.4)	1.11 (0.66–1.86)	
No	143 (90)	17.4 (8–24.5)	1.0	
Calcium				*p* = *0.36*
Yes	10 (6)	16.5 (11.2.21.4)	1.34 (0.7–2.56)	
No	149 (94)	17.3 (8.8–24.5)	1.0	
Ursodeoxy acid				*p* = *0.57*
Yes	13 (8)	23 (17.6–24.1)	0.85 (0.48–1.5)	
No	146 (92)	16.3 (8.5–24.5)	1.0	
Ferrofumerate				*p* = *0.25*
Yes	47 (30)	17.8 (11.8–26.2)	0.82 (0.58–1.16)	
No	112 (70)	16.5 (8–24.4)	1.0	

^a^In 56 cases data on medication use was not retrievable

### Multivariate analysis of factors related to the interval between IBD and CRC

**Fig. 4:**
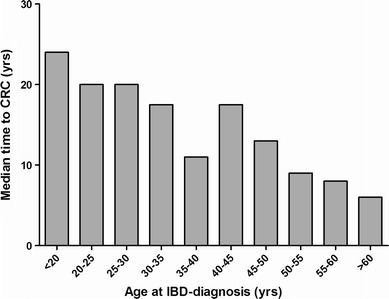
Age at IBD diagnosis is related to the time to develop CRC. This figure shows the time to develop CRC against the age at which patients were diagnosed with IBD. This shows that age at IBD diagnosis is related to the time to develop CRC

Age at onset IBD, rectal sparing (UC patients), colorectal surgery prior to onset IBD, and ongoing active inflammation during follow-up were assessed in a multivariate Cox-regression analysis. After correcting for possible confounders, age at onset of IBD (HR 10 years older age 2.29; 95 % CI 1.92–2.74, *p* < 0.001) and ongoing active inflammation during follow-up (HR 1.5; 95 % CI 1.0–2.29, *p* = 0.04) were significantly correlated with a shorter interval between IBD and CRC. Figure 4 demonstrates the relationship between age at IBD diagnosis and time to develop CRC.

## Discussion

We present a large nationwide general hospital study on the characteristics of IBD-related CRC. In this cohort we demonstrate early occurrence of IBD-related CRC and we show that a high proportion of IBD-associated CRCs develop before the recommended start of surveillance. Moreover, the patients who were included in a surveillance program had significantly better tumor characteristics. We identified a subgroup of patients in need of earlier start of surveillance.

A large number of patients (35 %) developed CRC in less than 8 years after diagnosis of IBD, which is before the recommended start of surveillance. Our results underline the results of two recently published academic cohorts, in which approximately 20 % of IBD-related tumors occurred within 10 years after IBD diagnosis [13, 14]. Probably, start of IBD-related symptoms instead of interval since diagnosis better estimates the years at risk for developing CRC; however, the actual date of onset of symptoms that were in fact related to IBD are difficult to retrieve and may lead to recall bias. In our cohort, in 170 out of 251 cases (68 %) data were lacking concerning the actual onset of IBD-related symptoms prior to the baseline diagnosis.

The guidelines for surveillance are currently being altered. The classic guidelines recommended to start bi-annual colonoscopy surveillance after 8–10 years for extensive colitis and 15–20 years for left-sided disease (from onset of symptoms) [17]. However, more recent guidelines suggest to perform a screening colonoscopy after 8–10 years of disease in all IBD patients, to assess the true microscopic extent of inflammation [18, 20]. Our data are in line with these adjustments, as location of IBD did not influence the time to develop CRC in our cohort.

However, more than one third of all cases occur before this newly recommended screening schedule. Diagnosis of IBD at older age is significantly related to early development of IBD-related CRC in our cohort. This has also been demonstrated in previous studies [10, 13]. In large contrast to the older age at which patients with early CRC were diagnosed with IBD, the median age at time of CRC diagnosis was equal compared with those who developed CRC >8 years after IBD diagnosis. This suggests that age at onset of IBD, and not duration of disease is a leading factor for early development of CRC in IBD patients. This could among others be explained by the fact that in UC patients colonocyte telomeres shorten with age almost twice as rapidly as in normal controls [23]. Telemore-shortening is associated with the development of cancer and colonocytes of UC patients show premature shortening of telomeres, which might in part explain the increased and earlier risk of cancer in this disease. Future research is needed to support this hypothesis. Another explanation might be that patients with late onset of IBD, similar to the general population, accumulate malignant risk factors until the onset of IBD [24].

Even though almost half of the patients with CRC had extensive colitis, the majority of IBD-related tumors were located in the left colon and already advanced tumor stages were found at time of diagnosis. More advanced tumors (AJCC stage III tumor) were found in our non-academic cohort (30 %) compared with the Dutch academic cohort (20 %). This might be explained by the fact that only 12 % of our patients were included in a dysplasia surveillance programme before development of CRC, against a slightly higher percentage (15 %) in the academic cohort. Surveillance colonoscopies are not yet common practice in general hospitals in the Netherlands and most gastroenterologists in the Netherlands do not adhere to surveillance guidelines [25]. This low adherence to colonoscopy surveillance in IBD patients reflects clinical practice in general hospitals with a presumed perception of low CRC risks in these populations. This perception has to change, as our findings support the inclusion of patients in surveillance strategies and show that surveillance was associated with earlier detection and thus improved tumor stage. Our findings are supported by previous studies, which also demonstrated that surveillance colonoscopies may detect CRC at an earlier stage and improve prognosis [15, 16]. Only recently, nationwide guidelines were launched for surveillance colonoscopies [26]. During our study period, surveillance was not regularly performed in the Netherlands. This underscores the need for an increased surveillance programme with an earlier start in the high risk group. However, more data are needed to establish proper surveillance methods in Crohn’s disease. For UC it has been estimated that 33 biopsy specimens are required to give 90 % confidence to detect dysplasia if it is indeed present [27]. However, the focal nature of inflammation in CD, the possibility of strictures and the prevalence of segmental resection means that surveillance practice in UC cannot be transferred directly to CD. The use of targeted biopsies, aimed at lesions identified by chromoendoscopy or endomicroscopy, has changed the policy of taking biopsies in UC and this policy should also be considered in patients with CD. However, more data are needed to establish a proper surveillance method in Crohn’s disease.

Strikingly, in 36 out of 251 patients (14 %), IBD and CRC were diagnosed simultaneously. All these patients were >37 years old at time of IBD diagnosis, which supports our notion that age at onset of IBD is probably a leading factor for early CRC. Most patients (15/21 patients, data missing in 15 cases) already had symptoms attributable to IBD before diagnosis. In the Dutch academic cohort 7 % of patients were diagnosed with IBD and CRC simultaneously [14]. However, this academic cohort also yielded a higher surveillance rate and they found less advanced tumors in their cohort. This might be explained by a difference in clinical practice between general hospitals and academic hospitals. This emphasizes the clinical relevance of our study to distinguish between several patients group, both in a academic setting as in the general population.

Inflammatory bowel disease patients with concomitant PSC and a positive family history of CRC are at increased risk for developing CRC [4, 7, 28–30]. However, we could not confirm that these factors also lead to earlier development of CRC, which is also endorsed by results from previous studies [13, 31, 32]. These results suggest that, although PSC and a familial predisposal of CRC are risk factors for acquiring CRC, they are not a driving force for earlier development of IBD-related CRC. It therefore remains a discussion whether we should perform surveillance colonoscopies more frequently in these patients. However, our results could be biased due to the relatively small number of patients with PSC in our non-academic cohort. Moreover, the number of patients with a positive family history of CRC might be under-recorded in a retrospective study. Therefore caution is needed in interpreting these results.

Use of medication and duration of medication use did not influence the interval between IBD and CRC in our cohort. Previous studies have demonstrated 5-ASA use to be protective for acquiring CRC [5, 33–35]. However, we could not confirm this in our cohort. This could be influenced by the retrospective data collection, in which duration of medication use was subdivided in four different periods and therefore not assessed as a continuous factor. Furthermore, medication use could have been prone to information bias due to the retrospective study design.

A recently published study from Sweden demonstrated that male patients have an overall 60 % higher rate of CRC than female patients [36]. However, there was no effect of sex on the RR within 10 years after IBD-diagnosis. Although we did not focus on gender differences, we can conclude that in our cohort also more male patients developed IBD-related CRC during our study-period of 15 years. However, we did not correct this for possible confounders as age and type of IBD. Although male patients were in the majority in our cohort, gender did not influence early development of CRC in our cohort. Although the Swedish study did not assess the time interval between IBD onset and CRC development, they could not identify a RR difference within 10 years of IBD, which supports our results.

In our study, eight out of 23 patients with a known left-sided UC developed a tumor in the right part of the colon. All patients had a well established IBD. However, it has been demonstrated that colonoscopy frequently underestimates the extent of colitis activity, as shown by histological evidence of colitis in macroscopically normal areas of the mucosa [37–40]. Therefore, the ECCO-guidelines recommend to perform a colonoscopy with histological biopsies in every IBD patient after 8 years of disease to establish the true extent of disease, even in those with only limited colitis [41]. However, in our study-period this was not yet common practice. This could explain why in eight out of 23 patients with a left-sided UC, the tumor was developed in the right part of the colon.

In our cohort, only 13 % of the cases were identified with dysplasia. However, our study was not primary designed to identify dysplasia. In order to determine how many cases of true dysplasia were identified during our study-period, we have searched the PALGA database for patients with true dysplasia in the 38 largest non-academic hospitals of the Netherlands. We identified 4436 patients suggestive for dysplasia and a possible diagnosis of IBD in their medical history. However, as in our study, many patients will be excluded after manual review of the search and reviewing clinical data in patient charts. Unfortunately, it was not feasible to verify all diagnoses in the hospital charts of this large group of patients all over the country. Moreover, a recent study has demonstrated that histological review of histological slides by a panel of experts reduced the total number of flat low-grade dysplasia patients with approximately 75 % [42].

In 2005, the Crude Incidence rate for CRC in the Netherlands was 66.73 per 100,000 persons [43]. This comprises all kinds of CRC, including IBD-related CRC. Earlier, we have demonstrated that the population rate for IBD-related CRC between 1990 and 2005 was approximately 0.05 % per year [44]. In a sub-analysis we excluded patients >65 years old to minimize possible interference with sporadic CRC. However, excluding this subgroup of patients did not influence our results with regard to early development of CRC. Moreover, all of our patients had a well-established IBD. Therefore we believe we maximally reduced the interference with sporadic CRC in our cohort.

Although we are aware of the limitations of a retrospective study design, this design was carefully chosen to fit our main aim to study the overall epidemiology of IBD-related CRC. Unfortunately not all data could be retrospectively recovered from the clinical charts, which is attributed to the retrospective study design. Because of the considerable time-interval to development of CRC in IBD and the low frequency of IBD-related CRC in general hospitals, conducting a prospective study would not be feasible. Using a retrospective analysis, we were able to perform a large nationwide survey with a follow-up time of 15 years.

In conclusion, our results emphasize the problem of a high proportion of IBD-associated CRCs developing before the recommended start of surveillance. Secondly, we demonstrate that surveillance is associated with a better tumor stage. This underlines the need to identify a subgroup of patients that require earlier start of surveillance. Based on our results, we suggest that older age at onset of IBD could be an additional factor to start surveillance in IBD patients. Accordingly, we would recommend earlier start of surveillance and an immediate start at time of IBD diagnosis for patients diagnosed with IBD above the age of 45 years. Moreover, better registration of start of symptoms is needed. Adapting the surveillance guidelines and identifying patients in need of earlier start of surveillance will detect CRC at an earlier stage and may therewith improve prognosis.

## References

[1] Eaden JA, Abrams KR, Mayberry JF (2001). The risk of colorectal cancer in ulcerative colitis: a meta-analysis. Gut.

[2] Eaden J (2004). Review article: colorectal carcinoma and inflammatory bowel disease. Aliment Pharmacol Ther.

[3] Jess T, Gamborg M, Matzen P, Munkholm P, Sorensen TI (2005). Increased risk of intestinal cancer in Crohn’s disease: a meta-analysis of population-based cohort studies. Am J Gastroenterol.

[4] Broome U, Lofberg R, Veress B, Eriksson LS (1995). Primary sclerosing cholangitis and ulcerative colitis: evidence for increased neoplastic potential. Hepatology.

[5] Velayos FS, Loftus EV, Jess T, Harmsen WS, Bida J, Zinsmeister AR (2006). Predictive and protective factors associated with colorectal cancer in ulcerative colitis: a case-control study. Gastroenterology.

[6] Rutter MD, Saunders BP, Wilkinson KH, Rumbles S, Schofield G, Kamm MA (2004). Cancer surveillance in longstanding ulcerative colitis: endoscopic appearances help predict cancer risk. Gut.

[7] Askling J, Dickman PW, Karlen P, Brostrom O, Lapidus A, Lofberg R (2001). Family history as a risk factor for colorectal cancer in inflammatory bowel disease. Gastroenterology.

[8] Rutter M, Saunders B, Wilkinson K, Rumbles S, Schofield G, Kamm M (2004). Severity of inflammation is a risk factor for colorectal neoplasia in ulcerative colitis. Gastroenterology.

[9] Ekbom A, Helmick C, Zack M, Adami HO (1990). Ulcerative colitis and colorectal cancer. A population-based study. N Engl J Med.

[10] Gyde SN, Prior P, Allan RN, Stevens A, Jewell DP, Truelove SC (1988). Colorectal cancer in ulcerative colitis: a cohort study of primary referrals from three centres. Gut.

[11] Sugita A, Sachar DB, Bodian C, Ribeiro MB, Aufses AH, Jr., Greenstein AJ. Colorectal cancer in ulcerative colitis. Influence of anatomical extent and age at onset on colitis-cancer interval. Gut. 1991;32:167–9. 10.1136/gut.32.2.167 PMC1378801 1864536

[12] Kvist N, Jacobsen O, Kvist HK, Norgaard P, Ockelmann HH, Schou G (1989). Malignancy in ulcerative colitis. Scand J Gastroenterol.

[13] Brackmann S, Andersen SN, Aamodt G, Langmark F, Clausen OP, Aadland E (2009). Relationship between clinical parameters and the colitis-colorectal cancer interval in a cohort of patients with colorectal cancer in inflammatory bowel disease. Scand J Gastroenterol.

[14] Lutgens MW, Vleggaar FP, Schipper ME, Stokkers PC, van der Woude CJ, Hommes DW (2008). High frequency of early colorectal cancer in inflammatory bowel disease. Gut.

[15] Choi PM, Nugent FW, Schoetz DJ, Silverman ML, Haggitt RC (1993). Colonoscopic surveillance reduces mortality from colorectal cancer in ulcerative colitis. Gastroenterology.

[16] Collins PD, Mpofu C, Watson AJ, Rhodes JM. Strategies for detecting colon cancer and/or dysplasia in patients with inflammatory bowel disease. Cochrane Database Syst Rev. 2006;CD000279. 10.1002/14651858.CD000279.pub3 16625534

[17] Eaden JA, Mayberry JF (2002). Guidelines for screening and surveillance of asymptomatic colorectal cancer in patients with inflammatory bowel disease. Gut.

[18] Winawer S, Fletcher R, Rex D, Bond J, Burt R, Ferrucci J (2003). Colorectal cancer screening and surveillance: clinical guidelines and rationale-update based on new evidence. Gastroenterology.

[19] Farraye FA, Odze RD, Eaden J, Itzkowitz SH (2010). AGA technical review on the diagnosis and management of colorectal neoplasia in inflammatory bowel disease. Gastroenterology.

[20] Biancone L, Michetti P, Travis S, Escher JC, Moser G, Forbes A et al. European evidence-based consensus on the management of ulcerative colitis: special situations. J Crohn’s Colitis. 2008;2:63–92. 10.1016/j.crohns.2007.12.001 21172196

[21] Casparie MK, Tiebosch ATMG, Burger G, Blauwgeers H, Pol van de A., van Krieken JHJM et al. Pathology databanking and biobanking in The Netherlands, a central role for PALGA, the nationwide histopathology and cytopathology data network and archive. Cell Oncol. 2007;29:19–24. 10.1155/2007/971816 PMC4618410 17429138

[22] Steyerberg EW, Eijkemans MJ, Harrell FE, Habbema JD (2000). Prognostic modelling with logistic regression analysis: a comparison of selection and estimation methods in small data sets. Stat Med.

[23] Risques RA, Lai LA, Brentnall TA, Li L, Feng Z, Gallaher J (2008). Ulcerative colitis is a disease of accelerated colon aging: evidence from telomere attrition and DNA damage. Gastroenterology.

[24] Brackmann S, Andersen SN, Aamodt G, Roald B, Langmark F, Clausen OP (2009). Two distinct groups of colorectal cancer in inflammatory bowel disease. Inflamm Bowel Dis.

[25] van Rijn AF, Fockens P, Siersema PD, Oldenburg B (2009). Adherence to surveillance guidelines for dysplasia and colorectal carcinoma in ulcerative and Crohn’s colitis patients in the Netherlands. World J Gastroenterol.

[26] CBO guideline for diagnosis and treatment of inflammatory bowel diseases in adult patients 2009. 2011.

[27] Rozen P, Baratz M, Fefer F, Gilat T (1995). Low incidence of significant dysplasia in a successful endoscopic surveillance program of patients with ulcerative colitis. Gastroenterology.

[28] Brentnall TA, Haggitt RC, Rabinovitch PS, Kimmey MB, Bronner MP, Levine DS (1996). Risk and natural history of colonic neoplasia in patients with primary sclerosing cholangitis and ulcerative colitis. Gastroenterology.

[29] Kornfeld D, Ekbom A, Ihre T (1997). Is there an excess risk for colorectal cancer in patients with ulcerative colitis and concomitant primary sclerosing cholangitis? A population based study. Gut.

[30] Shetty K, Rybicki L, Brzezinski A, Carey WD, Lashner BA (1999). The risk for cancer or dysplasia in ulcerative colitis patients with primary sclerosing cholangitis. Am J Gastroenterol.

[31] Nuako KW, Ahlquist DA, Sandborn WJ, Mahoney DW, Siems DM, Zinsmeister AR (1998). Primary sclerosing cholangitis and colorectal carcinoma in patients with chronic ulcerative colitis: a case-control study. Cancer.

[32] Lindberg BU, Broome U, Persson B (2001). Proximal colorectal dysplasia or cancer in ulcerative colitis. The impact of primary sclerosing cholangitis and sulfasalazine: results from a 20-year surveillance study. Dis Colon Rectum.

[33] van Staa TP, Card T, Logan RF, Leufkens HG (2005). 5-Aminosalicylate use and colorectal cancer risk in inflammatory bowel disease: a large epidemiological study. Gut.

[34] Velayos FS, Terdiman JP, Walsh JM (2005). Effect of 5-aminosalicylate use on colorectal cancer and dysplasia risk: a systematic review and metaanalysis of observational studies. Am J Gastroenterol.

[35] Chan EP, Lichtenstein GR (2006). Chemoprevention: risk reduction with medical therapy of inflammatory bowel disease. Gastroenterol Clin North Am.

[36] Soderlund S, Granath F, Brostrom O, Karlen P, Lofberg R, Ekbom A (2010). Inflammatory bowel disease confers a lower risk of colorectal cancer to females than to males. Gastroenterology.

[37] Kiesslich R, Fritsch J, Holtmann M, Koehler HH, Stolte M, Kanzler S (2003). Methylene blue-aided chromoendoscopy for the detection of intraepithelial neoplasia and colon cancer in ulcerative colitis. Gastroenterology.

[38] Kleer CG, Appelman HD (1998). Ulcerative colitis: patterns of involvement in colorectal biopsies and changes with time. Am J Surg Pathol.

[39] Riley SA, Mani V, Goodman MJ, Dutt S, Herd ME (1991). Microscopic activity in ulcerative colitis: what does it mean?. Gut.

[40] Geboes K, Ectors N, D’Haens G, Rutgeerts P (1998). Is ileoscopy with biopsy worthwhile in patients presenting with symptoms of inflammatory bowel disease?. Am J Gastroenterol.

[41] Biancone L, Michetti P, Travis S, Escher JC, Moser G, Forbes A (2008). European evidence-based consensus on the management of ulcerative colitis: special situations. J Crohns Colitis.

[42] Van Schaik et al. Substantial increase of progression rate of flat low-grade dysplasia in inflammatory bowel disease after review by an expert panel. Dutch Society of Gastroenterology & Hepatology (NVGE), Veldhoven, The Netherlands. 2010.

[43] http://www.cijfersoverkanker.nl. 2012.

[44] Baars JE, Looman CW, Steyerberg EW, Beukers R, Tan AC, Weusten BL et al. The risk of inflammatory bowel disease-related colorectal carcinoma is limited: results from a nationwide nested case-control study. Am J Gastroenterol. 2010;106(2):319–328. 10.1038/ajg.2010.428 21045815

